# Fasting-induced hormonal regulation of lysosomal function

**DOI:** 10.1038/cr.2017.45

**Published:** 2017-04-04

**Authors:** Liqun Chen, Ke Wang, Aijun Long, Liangjie Jia, Yuanyuan Zhang, Haiteng Deng, Yu Li, Jinbo Han, Yiguo Wang

**Affiliations:** 1 MOE Key Laboratory of Bioinformatics, Tsinghua-Peking Center for Life Sciences, School of Life Sciences, Tsinghua University, Beijing 100084, China; 2 MOE Key Laboratory of Bioinformatics, School of Life Sciences, Tsinghua University, Beijing 100084, China; 3 Key Laboratory of Nutrition and Metabolism, Institute for Nutritional Sciences, Shanghai Institutes for Biological Sciences, Chinese Academy of Sciences, University of Chinese Academy of Sciences, Shanghai 200031, China

**Keywords:** FGF21, fasting, lysosome, autophagy, lipid, TFEB

## Abstract

Lysosomes are centers for nutrient sensing and recycling that allow mammals to adapt to starvation. Regulation of lysosome dynamics by internal nutrient signaling is well described, but the mechanisms by which external cues modulate lysosomal function are unclear. Here, we describe an essential role of the fasting-induced hormone fibroblast growth factor 21 (FGF21) in lysosome homeostasis in mice. *Fgf21* deficiency impairs hepatic lysosomal function by blocking transcription factor EB (TFEB), a master regulator of lysosome biogenesis and autophagy. FGF21 induces mobilization of calcium from the endoplasmic reticulum, which activates the transcriptional repressor downstream regulatory element antagonist modulator (DREAM), and thereby inhibits expression of *Mid1* (encoding the E3 ligase Midline-1). Protein phosphatase PP2A, a substrate of MID1, accumulates and dephosphorylates TFEB, thereby upregulating genes involved in lysosome biogenesis, autophagy and lipid metabolism. Thus, an FGF21-TFEB signaling axis links lysosome homeostasis with extracellular hormonal signaling to orchestrate lipid metabolism during fasting.

## Introduction

Dynamic energy metabolism is tightly regulated by hormonal and nutritional signals. The dysfunction of regulatory signals and energy metabolism is linked to metabolic diseases such as non-alcoholic fatty liver disease and type 2 diabetes^1,2^. FGF21, a fasting-induced hormone with pleiotropic roles in energy metabolism, acts by binding its receptor FGFR1 and co-receptor β-Klotho (KLB)^3,4,5^. This promotes fatty acid oxidation, ketogenesis and gluconeogenesis in the liver, lipolysis in the adipose tissue, bone loss, and extension of the lifespan^3,4,5,6,7,8,9^. Although FGF21 is widely expressed in metabolic tissues such as liver, adipose tissues and pancreas, all circulating FGF21 is induced physiologically by fasting through peroxisome proliferator-activated receptor α (PPARα) predominantly in the liver^3,4,5,10,11,12^. In addition, FGF21 is also induced by ketogenic, amino acid-deficient and low-protein diets via PPARα and ATF4 (activating transcription factor 4)^4,10,13,14,15^. Pharmacologically, FGF21 and its analogs cause weight loss and improve the lipid profile and insulin sensitivity in obese mice, monkeys and humans^3,4,5,16^. All these results indicate that FGF21 is a master regulator that maintains energy homeostasis during adaptation to fasting, starvation or nutritional stress. Nevertheless, the mechanism by which FGF21 regulates lipid metabolism remains elusive.

The lysosome is a central organelle in energy metabolism and nutrient sensing and recycling in response to starvation or nutritional stress^1,17,18^. Lysosomes are also essential for autophagy, a catabolic process that helps to maintain cellular homeostasis and quality control^1,17,18,19,20^. In response to acute regulation of nutrient starvation or energy stress, mTOR (the mechanistic target of rapamycin) and AMPK (AMP-activated protein kinase) control autophagy flux^1,19,20^. Studies of the regulation of lysosome homeostasis have provided new insights into how the lysosomal-autophagic pathway adapts to starvation^17,18,21^. One of the master regulators, TFEB, controls the expression of an array of genes involved in lysosome biogenesis and autophagy^22,23^. When the organism is sufficiently fed, TFEB, a member of the basic helix-loop-helix leucine zipper family, is phosphorylated by mTOR and sequestered in the cytoplasm by binding to 14-3–3. In contrast, starvation or nutritional stress leads to dephosphorylation of TFEB, which shuttles to the nucleus and promotes the expression of genes in the CLEAR (coordinated lysosomal expression and regulation) network^17,24,25,26,27,28^. Recent studies demonstrated that the lysosomal-autophagic pathway plays a critical role in lipid metabolism by shuttling lipid droplets (LDs) to the lysosome for hydrolysis, a process named lipophagy^29^. In addition, TFEB has been shown to regulate lipid metabolism by promoting both lipophagy and lipid oxidation^30^. Although the effect of internal nutrition signaling on lysosome homeostasis has been extensively studied, it is unclear whether and how other external physiological stimuli affect lysosomal function.

Since both FGF21, an inter-organ long-range regulator of lipid metabolism, and the lysosomal-autophagic pathway, a local regulator of lipid metabolism, are adapted to starvation and exercise, we hypothesized that FGF21 may orchestrate lipid metabolism in concert with the lysosomal-autophagic pathway. Here, we show that FGF21 promotes TFEB-mediated lysosome biogenesis and lipophagy. FGF21 mobilizes calcium from the endoplasmic reticulum (ER) and activates the transcriptional repressor downstream regulatory element antagonist modulator (DREAM), thereby inhibiting expression of *Mid1*, the gene encoding the E3 ligase Midline-1. This leads to accumulation of protein phosphatase PP2A, a substrate of MID1. PP2A dephosphorylates TFEB, which enhances the expression of genes involved in lysosome biogenesis, autophagy and lipid metabolism. Thus, our results demonstrate that hormonal signaling, nutrient sensing and transcriptional regulation link lysosomal function to lipid metabolism through FGF21-TFEB signaling. This expands our understanding of the physiological regulation of the lysosomal-autophagic pathway and lipid metabolism, and provides insight into their related metabolic diseases.

## Results

### Impaired lysosomal function and enhanced lipid accumulation in *Fgf21*^−/−^ mice during fasting

**Figure 1:**
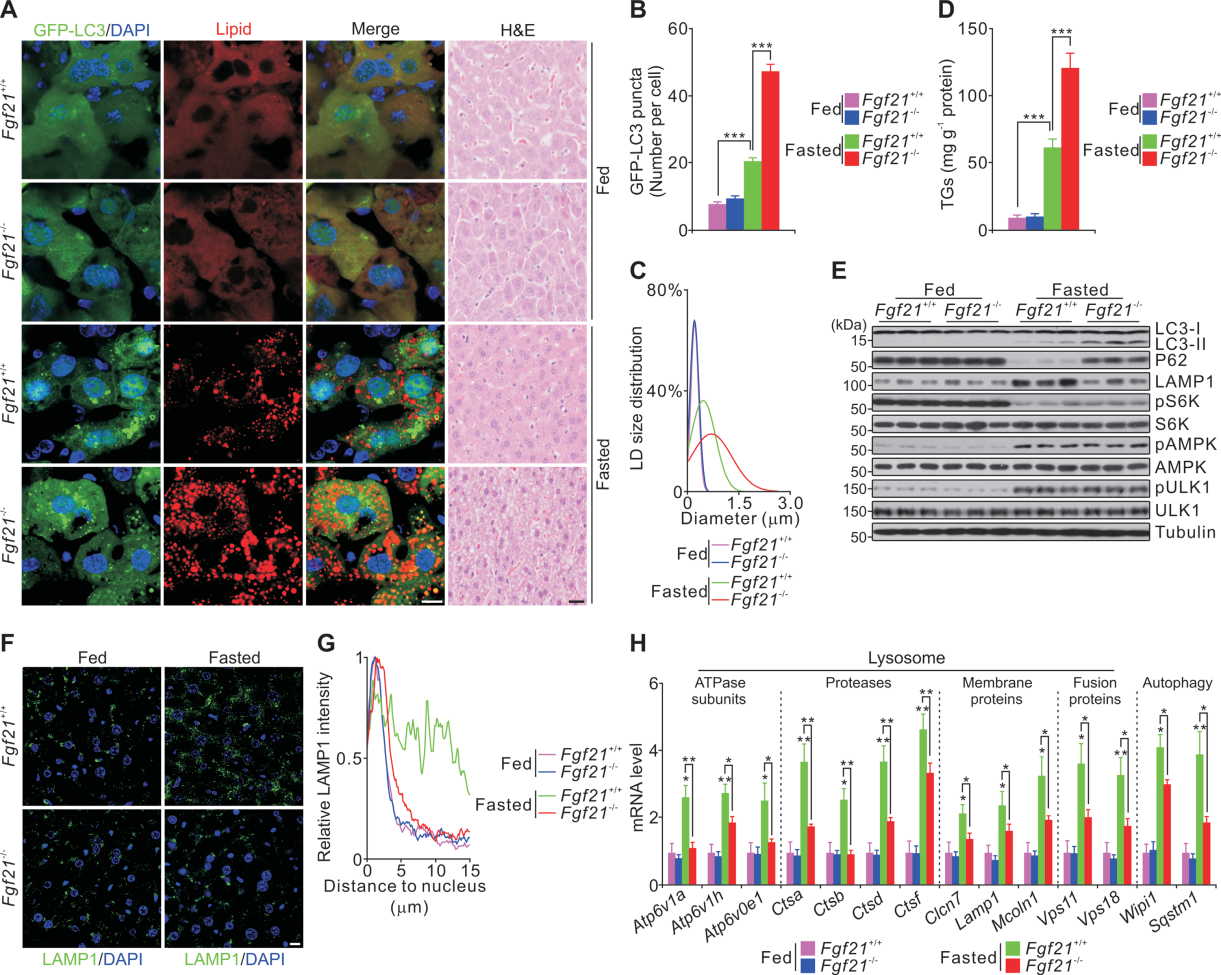
FGF21 deficiency impairs lysosomal function and enhances lipid accumulation in liver tissue. **(A-H)** Representative images **(A)**, quantification of GFP-LC3 puncta **(B)**, lipid droplet (LD) size **(C)**, levels of hepatic triglycerides (TGs) **(D)**, immunoblots **(E)**, LAMP1 staining **(F)** and quantification **(G)**, and qPCR results of the indicated genes **(H)** in liver extracts or sections from *Fgf21^+/+^* and *Fgf21*^−/−^ mice fed or fasted for 24 h. Scale bars, 10 μm. Data are shown as mean ± SEM. ^*^P < 0.05, ^**^P < 0.01, ^***^P < 0.001, *n* = 8 mice per group. DAPI, 4, 6-diamidino-2-phenylindole; H&E, hematoxylin and eosin.

To determine whether FGF21 regulates the lysosomal-autophagic pathway, we investigated the relationship between FGF21 levels and autophagy in fed mice and mice that were fasted for 12 h or 24 h. Fasted mice showed elevation of plasma FGF21 levels with concomitant enhancement of lipid oxidation (evaluated by quantitative PCR (qPCR) analysis of mRNA levels of genes involved in fatty acid β-oxidation) and autophagy (reflected by increased conversion of non-lipidated LC3-I (autophagy-induced light chain 3-I) to lipidated LC3-II and decreased P62 levels)^31^ in the liver (Supplementary information, Figure S1). This suggests a potential link between FGF21 and the lysosomal-autophagic pathway. Next, we tested hepatic triglyceride (TG) levels and autophagy in 24-week-old wild-type (WT) and *Fgf21*^−/−^ mice fed or fasted for 24 h, because FGF21 has a stronger effect on hepatic TG levels in older mice (Supplementary information, Figure S2). Autophagy (evaluated by numbers of GFP-LC3 puncta, LC3-II turnover and P62 levels), size and number of LDs, and levels of hepatic TGs were enhanced in the liver of WT mice after 24-h fasting (Figure 1A-1E), which is consistent with a previous report^29^. However, levels of LC3-II, P62 and TGs in *Fgf21*^−/−^ mice were much higher than those in WT animals after 24-h fasting, although the levels of autophagy markers and TGs were similar in fed WT and *Fgf21*^−/−^ mice (Figure 1A-1E). These results suggest that the lysosomal-autophagic pathway is blocked in fasted *Fgf21*^−/−^ mice.

Interestingly, both mTOR and AMPK activities were similar in WT and *Fgf21*^−/−^ mice under feeding or fasting conditions (Figure 1E). Together, the results suggest that FGF21 may affect lysosomal function, but not autophagy initiation. Consistent with this notion, lysosomal associated membrane protein 1 (LAMP1) levels (measured by immunoblotting and immunostaining) and mRNA levels of genes regulating lysosome biogenesis and autophagy were dramatically decreased in *Fgf21*^−/−^ mice compared to WT mice during fasting but not feeding (Figure 1E-1H). These results were further confirmed by adenoviral-mediated hepatic knockdown of *Klb* (Supplementary information, Figure S3), a co-receptor of FGF21^3,4,5^. Taken together, these results show that FGF21 promotes hepatic lysosomal function and lipid metabolism during fasting.

### TFEB mediates FGF21-enhanced lysosomal function

**Figure 2:**
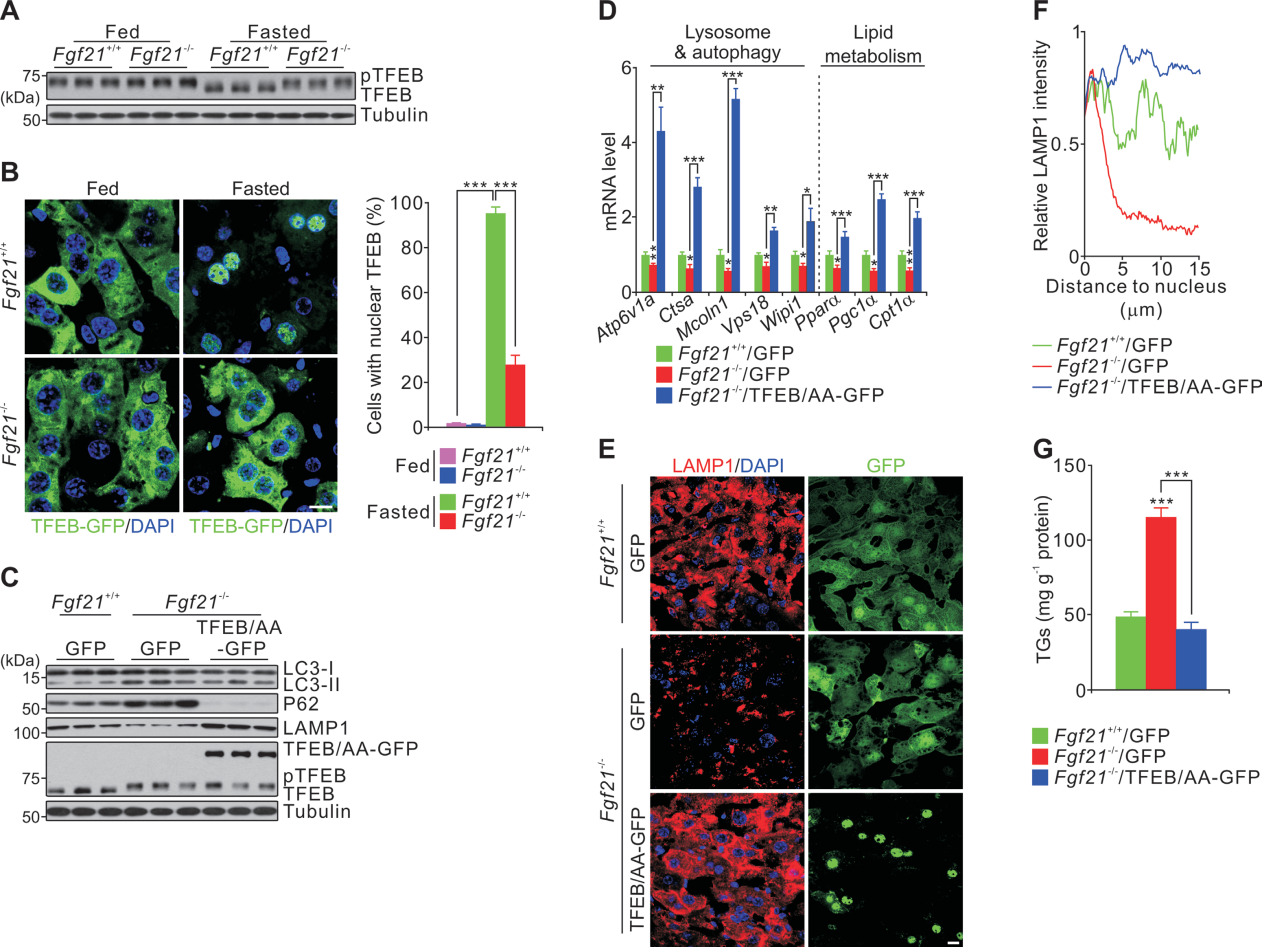
Hepatic nuclear activity of TFEB is reduced in *Fgf21*^−/−^ mice. **(A-B)** Immunoblots of liver extracts **(A)** and TFEB-GFP images **(B**, left panel**)** and quantification of nuclear translocation of TFEB **(B**, right panel**)** of frozen liver sections from *Fgf21^+/+^* and *Fgf21*^−/−^ mice fed or fasted for 24 h. **(C-G)** Immunoblots **(C)**, qPCR results **(D)**, LAMP1 staining **(E)** and quantification **(F)**, and hepatic triglyceride levels **(G)** showing the effect of hepatic overexpression of TFEB/AA-GFP (double alanine mutations at serine 142 and serine 211) in liver extracts or sections from *Fgf21*^−/−^ mice fasted for 24 h. Scale bar, 10 μm. Data are shown as mean ± SEM. ^*^P < 0.05, ^**^P < 0.01, ^***^P < 0.001, *n* = 8 mice per group.

TFEB, a master regulator of lysosome biogenesis and autophagy, regulates lipid metabolism at transcriptional levels^30^. mTOR-mediated phosphorylation of TFEB sequesters TFEB in the cytoplasm, whereas dephosphorylated TFEB shuttles to the nucleus and binds to the promoters of genes in the CLEAR network, thus promoting lysosome biogenesis and autophagy^17,21,22,23,24,25,26,27,28^. As lysosome biogenesis is impaired in *Fgf21*^−/−^ mice, we tested whether FGF21 affects TFEB activity. We examined TFEB band shifts on immunoblots, a well-acknowledged approach to assess TFEB phosphorylation^25,26,27,28^, and we directly visualized the subcellular localization of TFEB-GFP. In the fed state, TFEB is phosphorylated and sequestered in the cytoplasm in both WT and *Fgf21*^−/−^ mice (Figure 2A and 2B). In fasted WT animals, TFEB was dephosphorylated and relocated to the nucleus; however, in fasted *Fgf21*^−/−^ mice, TFEB was retained in the cytoplasm (Figure 2A and 2B).

Since nuclear-localized TFEB is activated to induce gene expression, we asked whether a mutant form of TFEB, that is retained in the nucleus, can rescue the defective lysosome biogenesis and lipid metabolism in *Fgf21*^−/−^ mice. To this end we made a TFEB mutant (TFEB/AA), which is confined to the nucleus because of serine-to-alanine mutations at positions 142 and 211 (Supplementary information, Figure S4), two major phosphorylation sites identified by previous reports^23,25,26,27,28^, and carried this in an adenoviral vector. Adenoviral-mediated hepatic overexpression of TFEB/AA-GFP completely restored lysosomal function and attenuated hepatic TG levels in fasted *Fgf21*^−/−^ mice (Figure 2C-2G). Taken together, these results indicate that TFEB acts downstream of FGF21 signaling to regulate lysosomal function.

### PP2A is necessary for FGF21-induced TFEB nuclear shuttling

**Figure 3:**
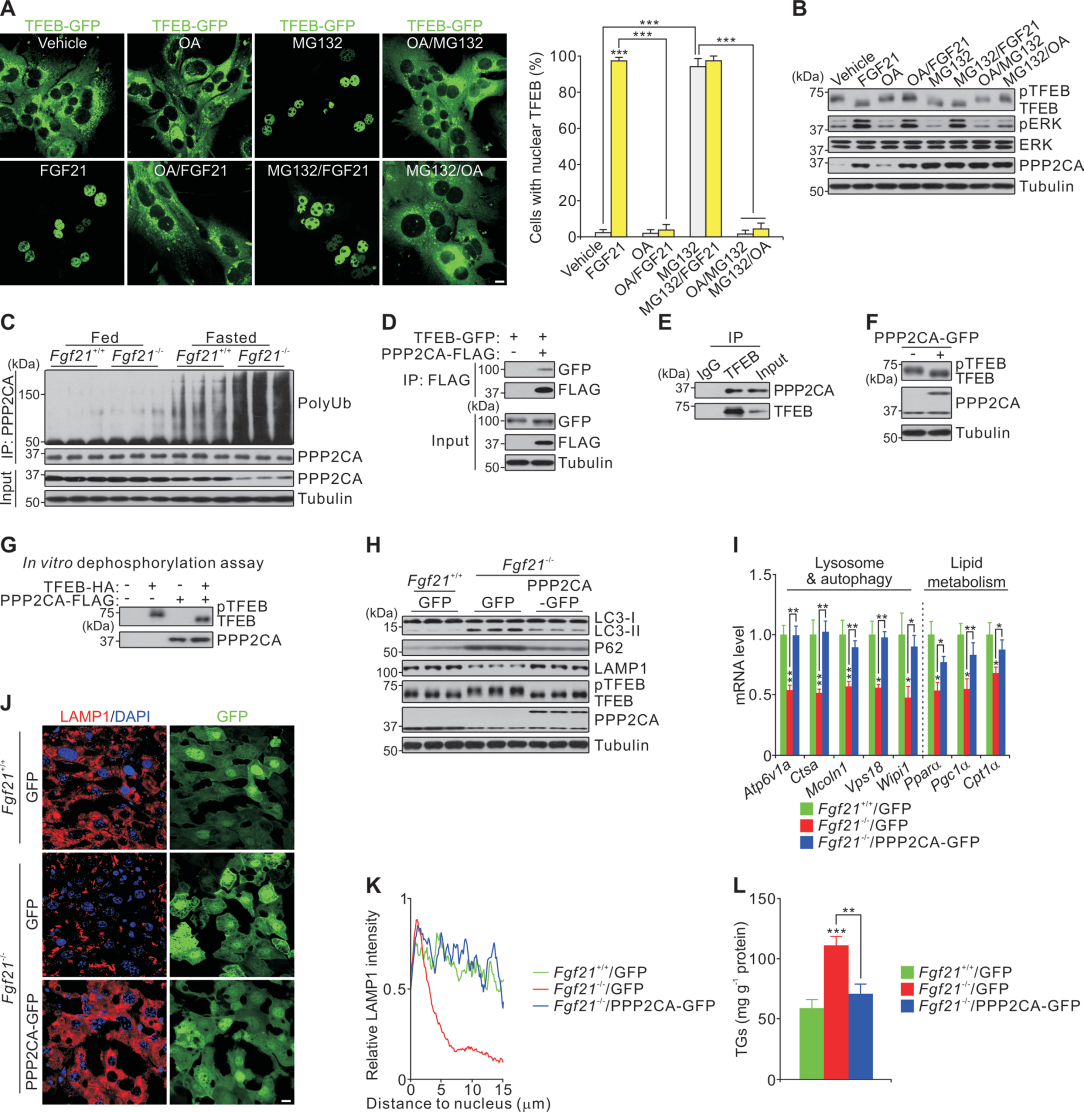
Protein phosphatase PP2A is required for FGF21-induced TFEB nuclear shuttling. **(A-B)** Representative images and quantification of nuclear translocation of TFEB **(A)** and immunoblots **(B)** showing the effect of different chemical treatments on cellular localization or phosphorylation of TFEB in mouse primary hepatocytes. Mouse primary hepatocytes were incubated with okadaic acid (OA, 100 nM) or MG132 (10 μM) for 1 h before 4 h FGF21 (50 ng/ml) stimulation or other treatment as indicated. OA/MG132, pretreatment with OA for 1 h before 4 h MG132 incubation; MG132/OA, pretreatment with MG132 for 1 h before 4 h OA incubation. **(C)** Immunoblots showing enhanced degradation of PPP2CA in liver extracts from *Fgf21*^−/−^ mice fasted for 24 h. **(D-E)** Co-immunoprecipitation showing the interaction of overexpressed **(D)** or endogenous **(E)** PPP2CA and TFEB in HEK293T cells **(D)** or mouse primary hepatocytes **(E)**. **(F-G)** Effect of PPP2CA on phosphorylation of TFEB by adenoviral PPP2CA-GFP overexpression in mouse primary hepatocytes **(F)** or by *in vitro* dephosphorylation assay **(G)**. The *in vitro* dephosphorylation assay was performed in PPP2CA-depleted cell lysates. Where indicated, eluates of immunoprecipitated TFEB-HA and/or PPP2CA-FLAG were added into the lysates and incubated for 30 min at 30 °C. **(H-L)** Adenoviral-mediated PPP2CA-GFP overexpression rescued the phenotypes in *Fgf21*^−/−^ mice fasted for 24 h. Immunoblots **(H)**, qPCR results **(I)**, LAMP1 staining **(J)** and quantification **(K)**, and hepatic triglyceride levels **(L)** in liver extracts or sections of the indicated groups. Scale bars, 10 μm. Data are shown as mean ± SEM. ^*^P < 0.05, ^**^P < 0.01, ^***^P < 0.001, *n* = 8 mice per group.

Although mTOR controls TFEB phosphorylation, no difference in mTOR activity was observed between WT and *Fgf21*^−/−^ mice (Figure 1E). It was recently reported that calcium released from lysosomes activates the protein phosphatase (PP) calcineurin, which then dephosphorylates TFEB^32^. Therefore, we tested whether phosphatases play an important role in determining the location of TFEB in mouse primary hepatocytes. The FGF21-induced dephosphorylation (of threonine 50, serine 142 and serine 211) and the nuclear translocation of TFEB is blocked by okadaic acid (OA), an inhibitor of PP1 and PP2A, but not by FK506 and Cyclosporin A (CsA), inhibitors of calcineurin (Figure 3A-3B and Supplementary information, Figure S5A-S5C). Interestingly, nuclear translocation of TFEB induced by Torin1 (an inhibitor of mTOR) is also blocked by OA pre-incubation (Supplementary information, Figure S5B). These results suggest that PP1 and/or PP2A are necessary for FGF21-induced TFEB activity, at least in primary hepatocytes. Strikingly, treatment with MG132 (a proteasome inhibitor) also resulted in dephosphorylation and nuclear shuttling of TFEB (Figure 3A and 3B). Moreover, the effect of MG132 treatment on TFEB was abolished by incubation with OA (Figure 3A and 3B). Taken together, these results demonstrate that FGF21 may regulate PP1 and/or PP2A to dephosphorylate TFEB in a proteasome-dependent manner.

To determine which phosphatase controls TFEB dephosphorylation, we tested the protein levels of different phosphatases in liver extracts from fed or fasted WT and *Fgf21*^−/−^ mice, and found that the amount of PPP2CA, a catalytic subunit of PP2A, was greatly reduced in fasted *Fgf21*^−/−^ mice (Figure 3C and Supplementary information, Figure S5D). PPP2CA was also ubiquitinated at much higher levels in fasted *Fgf21*^−/−^ mice than in fasted WT mice (Figure 3C). Co-immunoprecipitation assays of overexpressed proteins in HEK293T cells and endogenous proteins in mouse primary hepatocytes confirmed the interaction of PPP2CA with TFEB (Figure 3D and 3E). In addition, TFEB can be dephosphorylated *in vivo* following PPP2CA overexpression and *in vitro* following its incubation with purified PPP2CA (Figure 3F and 3G).

Based on these results, we hypothesized that PPP2CA overexpression would rescue the defects in lysosome and lipid metabolism in *Fgf21*^−/−^ mice. In support of this notion, hepatic lysosomal function and TG content in *Fgf21*^−/−^ mice with adenoviral-mediated overexpression of PPP2CA were all restored to levels similar to those in WT mice (Figure 3H-3L). Thus, PP2A mediates FGF21-regulated TFEB activity.

### The E3 ligase MID1 controls the level of PP2A

**Figure 4:**
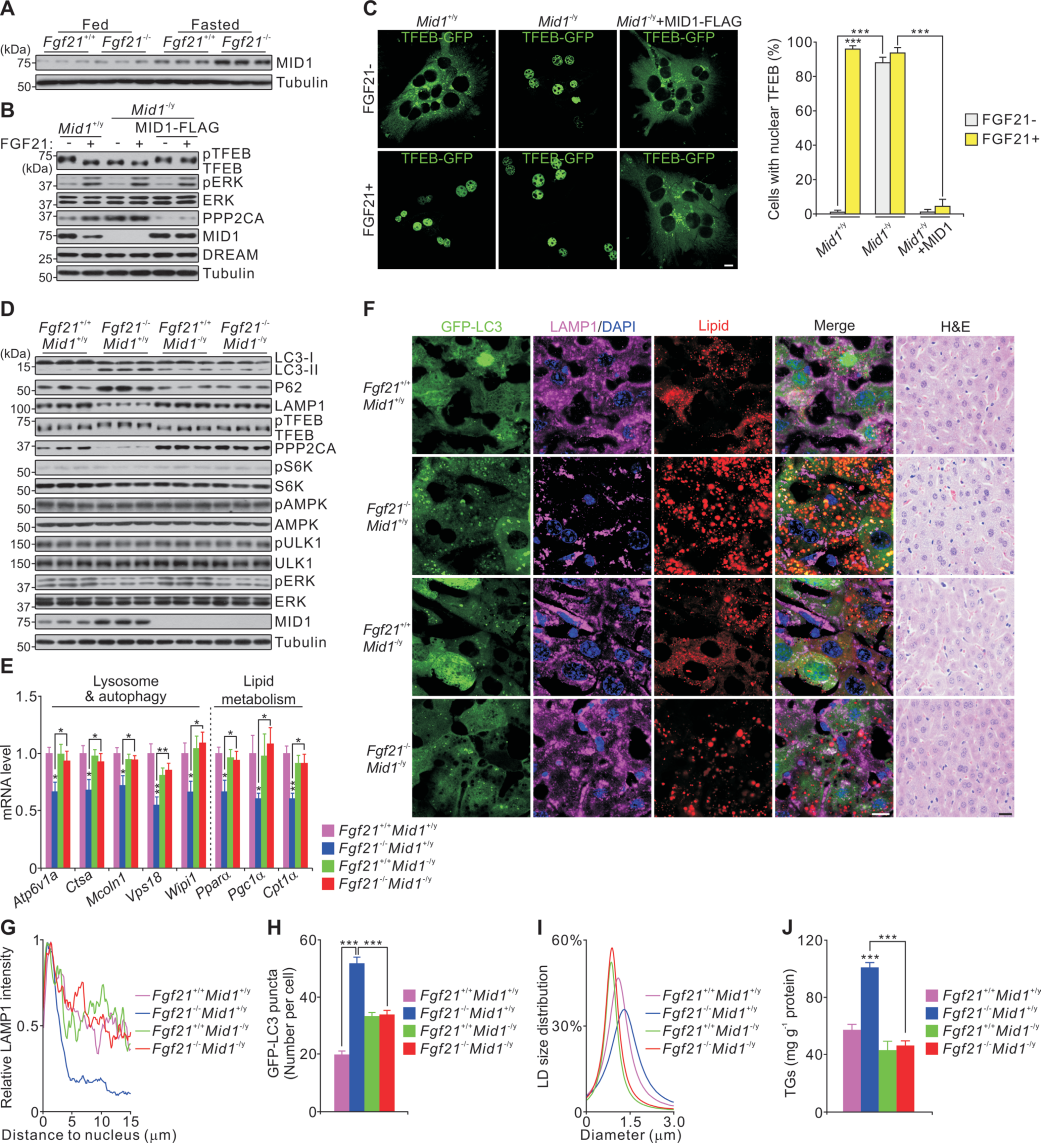
MID1 controls PPP2CA level and TFEB activity. **(A)** Immunoblots in liver extracts from *Fgf21^+/+^* and *Fgf21*^−/−^ mice fed or fasted for 24 h. **(B-C)** Effect of *Mid1* deficiency (*Mid1*^−^^/y^) on phosphorylation **(B)** and cellular localization **(C)** of TFEB. **(D-J**) Immunoblots **(D)**, qPCR results **(E)**, images **(F)**, LAMP1 intensity **(G)**, quantification of GFP-LC3 puncta **(H)**, LD size distribution **(I)** and hepatic triglyceride content **(J)** in liver extracts or sections from wild-type, *Fgf21*^−/−^, *Mid1*^-/y^ and *Fgf21^−/−^Mid1*^−/y^ mice fasted for 24 h. Scale bars, 10 μm. Data are shown as mean ± SEM. ^*^P < 0.05, ^**^P < 0.01, ^***^P < 0.001, *n* = 8 mice per group.

As PP2A regulates TFEB activity, we monitored the activity of a TFEB-targeted reporter (4 × CLEAR-Luc) to evaluate PP2A levels. To determine which E3 ligase controls PPP2CA stability, we performed a siRNA screen in which we knocked down individual genes and measured the effect on 4 × CLEAR-Luc activity in HepG2 cells (Supplementary information, Figure S6A). We focused upon three E3 ligases, UBR5, ASB3 (ankyrin repeat and SOCS box protein 3) and MID1, for further study because their deficiency enhanced luciferase activity (Supplementary information, Figure S6A and Table S1). The level of MID1, but not UBR5 or ASB3, in the liver was dramatically increased in *Fgf21*^−/−^ mice compared with WT mice during fasting (Figure 4A and Supplementary information, Figure S6B). In addition, the dramatic increase in the level of MID1 isoform 2, a major isoform in the liver, resulted from enhanced transcriptional expression rather than post-translational regulation (Supplementary information, Figure S6B-S6D).

Mutations in the *MID1* gene are causally linked to X-linked Opitz BBB/G syndrome, which primarily affects the ventral midline^33^. MID1 is known to be an E3 ligase that targets the catalytic subunit of PP2A for ubiquitin-mediated degradation^33^. We therefore investigated whether MID1 modulates PP2A levels and TFEB activity in mouse primary hepatocytes. *Mid1* deficiency increases PPP2CA levels and nuclear translocation of TFEB even in the absence of FGF21, and this effect is reversed by addition of MID1-FLAG (Figure 4B and 4C). Together, these results indicate that MID1 is an E3 ligase of PPP2CA, as previously reported^33^, and that MID1 is required for TFEB activity.

As MID1 level is increased in *Fgf21*^−/−^ mice and MID1 negatively regulates PP2A levels and TFEB activity, we tested whether *Mid1* knockout can restore the defective lysosome phenotype in *Fgf21*^−/−^ mice. After 24 h of fasting, *Mid1*-deficient animals had slightly improved lysosomal function and TG content in liver tissues compared to fasted WT mice (Figure 4D-4J). More impressively, double knockout (*Fgf21^−/−^Mid1*−/y) mice had similar phenotypes to WT mice (Figure 4D-4J), indicating that *Mid1* deficiency restored TFEB activity in a PP2A-dependent manner. These results were further confirmed by adenoviral-mediated hepatic knockdown of *Mid1* in *Fgf21*^−/−^ mice (Supplementary information, Figure S6E-S6I). Together, these results indicate that the E3 ligase MID1 downregulates PP2A levels and thereby inhibits TFEB activity.

### The transcriptional repressor DREAM suppresses *Mid1* expression

**Figure 5:**
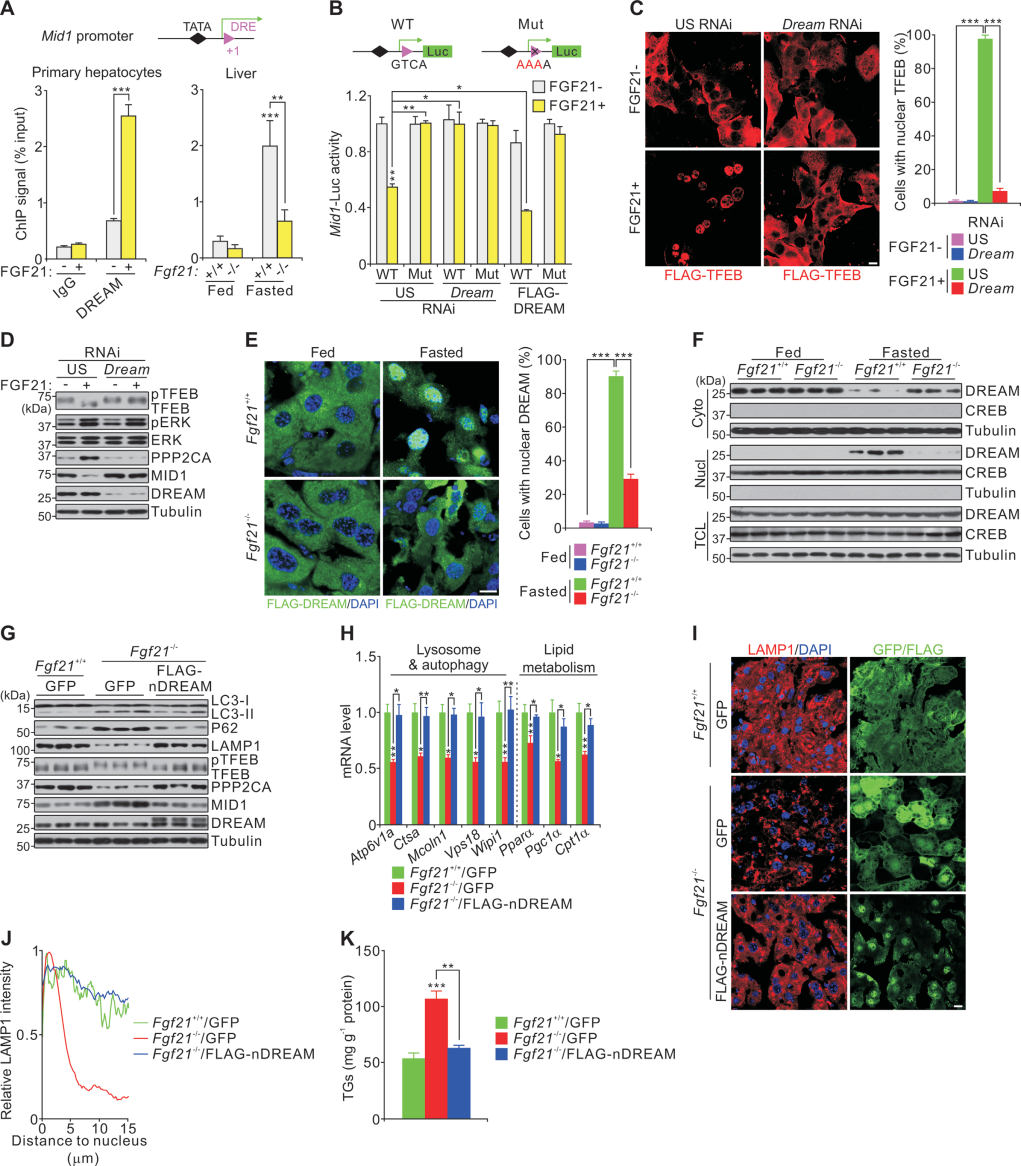
DREAM suppresses *Mid1* expression. **(A)** Location of the TATA box of the mouse *Mid1* gene, the transcriptional start site (green arrow) and the DRE site within exon 1 (top panel), and chromatin immunoprecipitation (ChIP) showing the occupancy of the *Mid1* promoter by DREAM in mouse primary hepatocytes in presence or absence of FGF21 (bottom left) and in liver extracts from *Fgf21^+/+^* and *Fgf21*^−/−^ mice fed or fasted for 24 h (bottom right). **(B)** Location of the wild-type (WT) and mutant (Mut) DRE site in the *Mid1*-Luc reporter (top), and the effect of FLAG-DREAM overexpression or *Dream* knockdown on the WT or Mut reporter in presence or absence of FGF21 in mouse primary hepatocytes (bottom). **(C-D)** Effect of *Dream* knockdown on cellular localization **(C)** and phosphorylation **(D)** of TFEB in presence or absence of FGF21 in mouse primary hepatocytes. **(E-F)** Cellular localization of DREAM evaluated by immunostaining of FLAG-DREAM in frozen liver sections **(E)** and immunoblots of liver extracts after subcellular fractionation **(F)** from *Fgf21^+/+^* and *Fgf21*^−/−^ mice fed or fasted for 24 h. **(G-K)** Immunoblots **(G)**, qPCR results **(H)**, LAMP1 staining **(I)** and quantification **(J)**, and hepatic triglyceride content **(K)** showing the effect of overexpression of nuclear-localized mutated DREAM (nDREAM, triple alanine mutations at serine 123, aspartic acid 251 and asparagine 253) in liver extracts or sections from *Fgf21*^−/−^ mice fasted for 24 h. Scale bars, 10 μm. Data are shown as mean ± SEM. ^*^P < 0.05, ^**^P < 0.01, ^***^P < 0.001, *n* = 8 mice or five replicates for hepatocytes per group.

On the basis of the finding that FGF21 negatively regulates *Mid1* expression, we speculated that FGF21 activates a transcriptional repressor to inhibit *Mid1* expression. The results from truncated reporter assays and calcium-dependent activity of *Mid1*-Luc further suggest that this repressor binds the *Mid1* promoter close to the transcription start site in a calcium-dependent manner (Supplementary information, Figure S7A and S7B). DREAM, also named Calsenilin or KChIP3, is a transcriptional repressor that binds specific DNA sequence elements (DREs) close to the transcriptional start site in exon 1 of *Pdyn* (prodynorphin) and c-*Fos*^34,35^. Calcium promotes transport of DREAM into the nucleus^36^. Interestingly, exon 1 of mouse *Mid1* contains one DRE just downstream of the transcription start site (Figure 5A, top panel). FGF21 treatment promotes DREAM nuclear translocation and occupancy at this *Mid1* DRE, as judged by chromatin immunoprecipitation (ChIP) assays in mouse primary hepatocytes (Figure 5A, left panel; Supplementary information, Figure S7C). These results were further confirmed in liver tissues from WT mice fasted for 24 h (Figure 5A, right panel). Occupancy of the *Mid1* DRE by DREAM was dramatically decreased in 24-h fasted *Fgf21*^−/−^ mice compared with WT mice (Figure 5A, right panel). This indicates that FGF21 is necessary to induce nuclear shuttling of DREAM, which thereby inhibits *Mid1* transcription during fasting. These results were further confirmed by luciferase-based reporter assays, as a mutated DRE (Mut) reporter, but not the WT reporter, lost the ability to respond to FGF21 (Figure 5B and Supplementary information, Figure S7D). Overexpression of DREAM further attenuated *Mid1*-Luc activity, whereas knockdown of *Dream* blunted the FGF21-induced repression of *Mid1*-Luc activity (Figure 5B and Supplementary information, Figure S7D). When *Dream* was knocked down in mouse primary hepatocytes, FGF21 failed to decrease the level of MID1, increase the level of PPP2CA or reduce the level of phosphorylated TFEB (Figure 5C and 5D). Collectively, these results demonstrate that DREAM's nuclear shuttling is required for FGF21-induced TFEB nuclear activity.

As nuclear shuttling of DREAM is critical for regulating TFEB activity, we investigated DREAM localization in WT and *Fgf21*^−/−^ mice by immunostaining and immunoblotting following subcellular fractionation. DREAM traveled from the cytoplasm to the nucleus after 24-h fasting in WT mice, whereas the shuttling was dramatically attenuated in *Fgf21*^−/−^ mice (Figure 5E and 5F). Considering the effect of nuclear DREAM on TFEB activity, we made a DREAM mutant (nDREAM), which constitutively binds to DRE. We did this by introducing three mutations (serine-to-alanine at position 123, aspartic acid-to-alanine at position 251 and asparagines-to-alanine at position 253). These mutations abolish the calcium responsiveness of DREAM and prevent it from dissociating from DNA and shuttling to the cytoplasm^34,37^ (Supplementary information, Figure S7E and S7F). Strikingly, nDREAM alone is sufficient to sequester TFEB in the nucleus (Supplementary information, Figure S7E and S7F). Furthermore, hepatic nDREAM overexpression decreased MID1 levels, increased PPP2CA levels and restored TFEB-mediated lysosomal function and lipid metabolism in *Fgf21*^−/−^ mice (Figure 5G-5K). Together, these results indicate that DREAM mediates FGF21-induced repression of *Mid1* by directly repressing the transcription of *Mid1* and thereby enhancing PPP2CA levels and TFEB activity.

### FGF21 enhances calcium release from the ER and nuclear shuttling of DREAM

**Figure 6:**
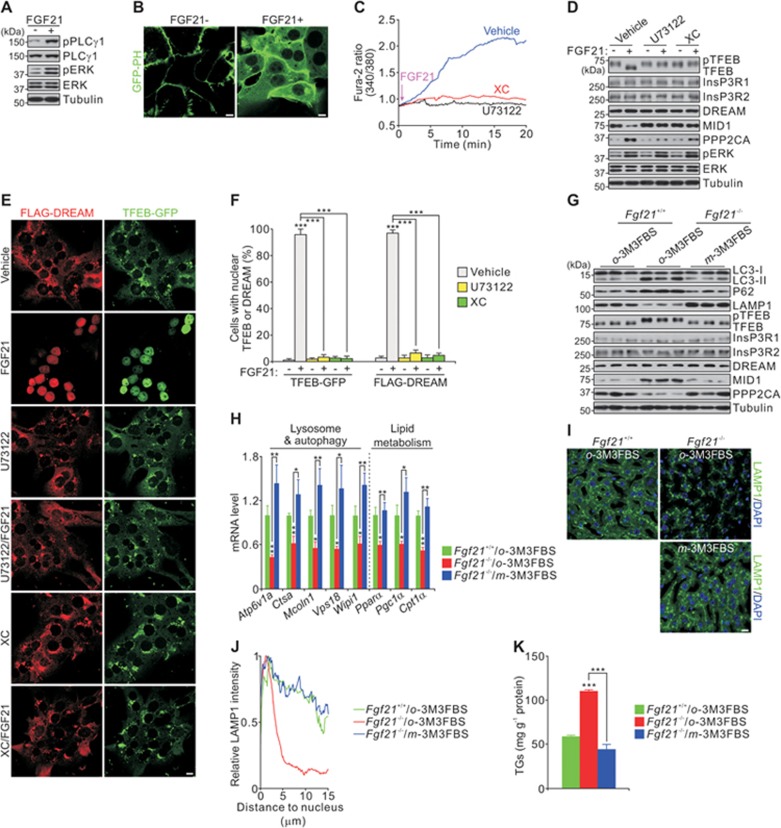
Enhanced nuclear translocation of DREAM and TFEB by FGF21-mobilized calcium. **(A-B)** FGF21 stimulation results in PLCγ1 activation measured by protein levels of tyrosine-phosphorylated PLC1 **(A)** and GFP-PH movement **(B)**. Mouse primary hepatocytes were incubated in presence or absence of FGF21 for 30 min. **(C)** Effect of FGF21 on calcium mobilization in presence or absence of U73122 (10 μM) or Xestospongin C (XC, 2 μM) in mouse primary hepatocytes. **(D-F)** Effect of U73122 and XC on TFEB phosphorylation **(D)**, the cellular localization of FLAG-DREAM and TFEB-GFP **(E)**, and quantification of nuclear translocation of TFEB or DREAM **(F)** in mouse primary hepatocytes. **(G-K)** Immunoblots **(G)**, qPCR results **(H)**, LAMP1 staining **(I)** and quantification **(J)**, and hepatic triglyceride content **(K)** showing the effect of *m*-3M3FBS in liver extracts or sections from *Fgf21*^−/−^ mice fasted for 24 h. *o*-3M3FBS is an inactive analog of *m*-3M3FBS used as a negative control. Scale bars, 10 μm. Data are shown as mean ± SEM. ^*^P < 0.05, ^**^P < 0.01, ^***^P < 0.001, *n* = 8 mice per group.

Phospholipase C γ1 (PLCγ1), when activated by FGFR, converts phosphatidylinositol-4,5-bisphospate (PIP2) into diacylglycerol and 1,4,5-triphosphate (InsP3), whereas InsP3 binds to its receptor InsP3R to mobilize calcium from the ER to the cytoplasm^38,39,40^. On the basis of these facts, we checked whether FGF21 is able to activate PLCγ1. By immunoblotting, we showed that FGF21 treatment increased the level of Tyr783-phosphorylated PLCγ1 (an active form of PLCγ1)^39,40^ (Figure 6A). We also used a GFP-PH domain assay, which is based on the observation that GFP-PH binds PIP2 and is mainly concentrated at the plasma membrane under basal conditions, but binds to InsP3 with much higher affinity and moves to the cytoplasm after PLCγ1 activation^39,40,41^. FGF21 treatment increased the cytoplasmic GFP-PH intensity (Figure 6B and Supplementary information, Figure S8A and S8B). Together, these results indicate that FGF21 can indeed activate PLCγ1. Decreased tyrosine phosphorylation of PLCγ1 in liver extracts of fasted *Fgf21*^−/−^ mice further confirmed the activating effect of FGF21 on PLCγ1 (Supplementary information, Figure S8C). In addition, FGF21 induced a slower and sustained calcium release in a PLCγ1- and InsP3R-dependent manner, because incubation with inhibitors (U73122 for PLC and XC for InsP3R) abrogated FGF21-induced calcium mobilization (Figure 6C). These inhibitors also reduced nuclear translocation of DREAM, increased the level of MID1 and then decreased the level of PPP2CA, which attenuated nuclear shuttling of TFEB (Figure 6D-6F). Compared to FGF21, glucagon (Gcg) stimulated a much more rapid and transient calcium pulse (Supplementary information, Figure S8D). Gcg, but not FGF21, activated calcineurin, as measured by dephosphorylation of CRTC2 and NFAT-Luc activity (Supplementary information, Figure S8E and S8F), as both NFAT and CRTC2 are substrates of calcineurin^42,43,44,45^.

As active PLCγ1 is reduced in *Fgf21*^−/−^ mice during fasting, we investigated whether *m*-3M3FBS (a PLCγ1 activator) can rescue the defective lysosome phenotype in *Fgf21*^−/−^ mice. Indeed, hepatic lysosome biogenesis and TG content in fasted *Fgf21*^−/−^ mice were all restored to levels similar to those in WT mice (Figure 6G-6K and Supplementary information, Figure S8G). Taken together, these results demonstrate that an increase in cytosolic calcium levels, modulated by an FGF21-PLCγ1-InsP3R axis, promotes DREAM-dependent TFEB nuclear shuttling.

## Discussion

**Figure 7:**
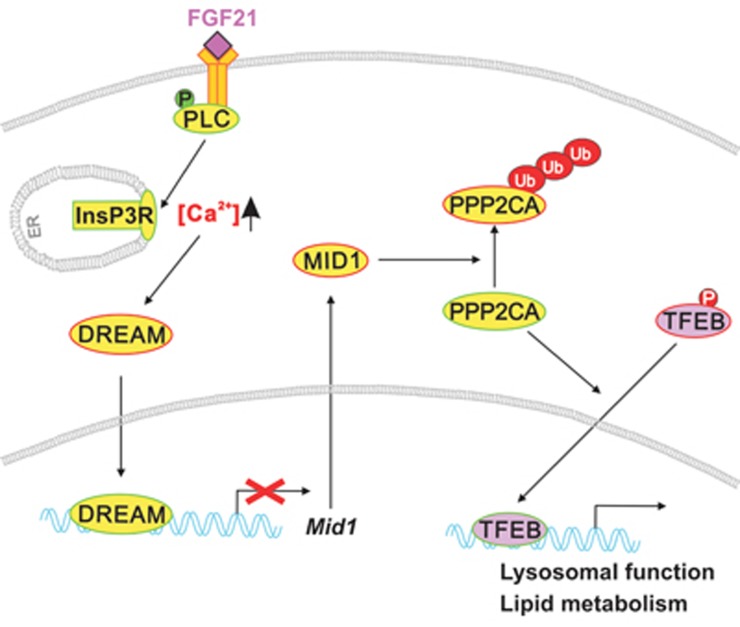
A model of coordinated lysosomal function and lipid metabolism mediated by FGF21. FGF21 induces calcium release from the ER in a PLCγ1- and InsP3R-dependent manner, which promotes DREAM nuclear translocation and thus inhibits *Mid1* expression. As a result, PPP2CA is stabilized and enhances TFEB dephosphorylation and nuclear shuttling. Activated TFEB then promotes the expression of target genes involved in lysosome biogenesis, autophagy and lipid metabolism.

During fasting, dynamic lipid metabolism in the liver is coordinated by inter-organ communication through hormones such as glucagon and FGF21, and by local nutrition-controlled process such as the lysosomal-autophagic pathway. However, the mechanisms that orchestrate hepatic lipid metabolism by linking inter-organ communication to local nutrient signaling are not well understood. This study demonstrates that the fasting-induced hormone FGF21 mobilizes calcium from the ER in a PLCγ1- and InsP3R-dependent manner, thus shuttling DREAM to the nucleus. DREAM then inhibits expression of the *Mid1* gene, leading to stabilization and accumulation of PP2A, which activates TFEB and enhances transcription of genes involved in lysosome biogenesis, autophagy and lipid metabolism (Figure 7).

Although it is controversial whether the liver is a direct site of FGF21 action, administration of a high dose of FGF21 in normal mice or obese mammals indeed affects hepatic downstream signaling of FGF21 and energy metabolism^3,4,5,16,46,47,48^, which suggests that hepatocytes are not sensitive to low doses of FGF21. The response to FGF21 might be affected by the different ages of the mice or different feeding or fasting schedules. A previous report^49^ showed that FGF21 deficiency has a slight effect on hepatic lipid metabolism in 8-week-old mice, whereas our results demonstrated that FGF21 deficiency in 24-week-old mice resulted in increased hepatic lipid accumulation (Supplementary information, Figure S2), suggesting that age enhances the effect of FGF21 on the hepatic lysosomal-autophagic pathway. It is possible that ageing promotes lipolysis in adipose tissue and lipid accumulation in the liver^50,51^, thereby activating the lysosomal-autophagic pathway, whereas the effect is not obvious in young mice without challenge. Of note, fasting slightly increases the level of MID1 and decreases the level of PPP2CA (Figures 3C and 4A), despite that it also results in DREAM translocation into the nucleus (Figure 5E). This suggests that other factors may regulate *Mid1* expression during early fasting independently of DREAM.

It is known that PPARα and TFEB control autophagy and/or lipophagy at the transcriptional level^23,30,52^. FGF21 is induced by PPARα after prolonged fasting or autophagy deficiency^10,11,12,53^, and our results now demonstrate that FGF21 promotes TFEB-targeted expression of genes involved in lysosome biogenesis, autophagy and lipid metabolism including *Pparα* and its coactivator *Pgc1α*. Thus, a feed-forward loop including PPARα, PGC1α, FGF21 and TFEB may orchestrate lipid metabolism by coordinating lysosome biogenesis, autophagy, lipid oxidation and mitochondrial function across different tissues or organs including skeletal muscle, liver and adipose tissue.

Adaption to fasting or starvation has evolved in animals to ensure the availability and conservation of energy when nutrients are scarce. The lysosomal-autophagic pathway plays a critical role in maintaining energy homeostasis during nutrient deprivation. Acute regulation of this pathway by signaling mechanisms linked to nutrient sensing has been well described and mTOR is a master regulator of this pathway^1,19,20^. Previous studies show that mTOR inhibition is sufficient to induce nuclear shuttling of TFEB without inhibition of phosphatase activity^25,26,27,28^, whereas our results demonstrate that PP2A modulates the FGF21-TFEB signaling axis during fasting. As the nuclear-localized TFEB is dephosphorylated, it is possible that both phosphatase activation and kinase inactivation are necessary to induce the rapid and maximal nuclear shuttling of TFEB and thereby to mediate the lysosomal-autophagic pathway in response to different environmental cues^32,54^.

Previous studies showed that FGF21 and MID1/PP2A affect mTOR activity during re-feeding, overfeeding or in feeding a high-fat diet^55,56,57^. However, mTOR activity is not affected by the FGF21-MID1/PP2A signaling axis after 24-h fasting, which is probably because of the very low activity of mTOR after fasting compared to feeding (Supplementary information, Figure S1B). During prolonged fasting, mTOR is reactivated by accumulated amino acids, which are generated by the starvation-induced lysosomal-autophagic pathway^19,20,58^. Activated mTOR then re-phosphorylates TFEB and attenuates its activity. In addition, endocytosis of FGFR^59^, which decreases its response to ligands, and dephosphorylation of InsP3R^38^, which decreases calcium mobilization, may also contribute to the attenuation of signaling. Together, these mechanisms guarantee a negative feedback to modulate the duration of FGF21-TFEB signaling.

There is controversy about the roles of calcium and InsP3R in the lysosomal-autophagic pathway, because different treatments produce conflicting results in different cell types under physiological conditions^60^. The outcome in each cell type depends on the different signaling inputs and the spatio-temporal characteristics of the calcium signals, including frequency, amplitude and duration. Our previous results showed that glucagon mobilizes rapid and transient calcium flux through InsP3R to activate calcineurin^44^, whereas calcium release from lysosomes through MCOLN1 (Mucolipin 1) also activates calcineurin and thereby promotes TFEB dephosphorylation and nuclear activity^32^. However, the slower and sustained calcium flux that occurs via PLCγ and InsP3R in response to FGF21 appears to have no effect on calcineurin (Supplementary information, Figure S8E and S8F). This suggests that the duration and amplitude of the calcium pulse are critical for signal specificity. The dependence of TFEB nuclear translocation on DREAM further excludes the possibility that calcineurin mediates nuclear shuttling of TFEB induced by FGF21 (Figure 5 and Supplementary information, Figure S7). In addition, calcineurin dephosphorylates TFEB at serine 142 and serine 211, whereas PP2A dephosphorylates TFEB at one extra site (threonine 50) (Supplementary information, Figure S5A and S5C). Although we have shown that FGF21 promotes TFEB activity via PP2A after prolonged fasting, we cannot exclude a possible role of calcineurin-mediated TFEB activity during early fasting, as Gcg promotes ER calcium mobilization via InsP3R and calcineurin activation^44^ (Supplementary information, Figure S8D-S8F). Together the results suggest that PP2A and calcineurin may discriminate different signaling inputs during early and late fasting.

Defective functioning of the lysosomal-autophagic pathway, dysregulated lipid metabolism and FGF21 resistance affect each other and may further exacerbate vulnerable energy homeostasis and insulin sensitivity in obesity^2,4,5,61^. Taken together, our results reveal novel mechanisms by which an FGF21-TFEB signaling axis integrates extracellular and intracellular signals to control lysosomal function. Our study expands our knowledge of the physiological regulation of lysosome homeostasis and may have implications for understanding the mechanisms underlying the lysosomal-autophagic pathway and lipid metabolism, and their related metabolic diseases.

## Materials and Methods

### Reagents

FGF21 (50 ng/ml, R&D), Gcg (100 nM, Sigma), okadaic acid (OA, 100 nM, Tocris Bioscience), MG132 (10 μM, Selleck), Cyclosporin A (CsA, 10 μM, Selleck), FK506 (5 μM, Selleck), A23187 (5 μM, Selleck), U73122 (5 μM, Selleck), *o*-3M3FBS (10 μM, TRC), *m*-3M3FBS (10 μM, TRC) and Xestospongin C (XC, 2 μM, Cayman Chemical) were used in this study. For single reagent treatments, mouse primary hepatocytes were incubated for 4 h unless indicated otherwise. For double reagent assays, hepatocytes were pretreated for 1 h with one reagent and then further incubated with the second one for another 4 h. Phosphorylated ERK (pERK) was used to evaluate FGF21 activity after 30 min incubation in mouse primary hepatocytes.

### Plasmids and adenoviruses

FLAG-tagged TFEB (FLAG-TFEB) and 4 × CLEAR-Luc plasmids were kindly provided by Dr Andrea Ballabio (Telethon Institute of Genetics and Medicine, Italy). PPP2CA-FLAG (CH808822) was purchased from ViGene Biosciences. DREAM (NM_001291005) was amplified from a mouse brain cDNA library. The PH domain of PLCδ1 (NM_006225) was amplified from a human kidney cDNA library. *PDYN*-Luc was cloned from the human *PDYN* promoter spanning from −185 to +375. *Mid1*-Luc was made from the mouse *Mid1* promoter spanning from −1 210 to +30. Site-directed mutagenesis was performed with the QuikChange strategy (Agilent Technologies).

Adenoviruses (2 × 10^8^ plaque forming units (pfu) carrying GFP, GFP-LC3, TFEB/AA-GFP, PPP2CA-FLAG, FLAG-nDREAM, *Mid1* shRNA, *Klb* shRNA^55^ or unspecific shRNA) were delivered to 24-week-old male WT, *Fgf21*^−/−^, *Mid1*^−/y^ or *Fgf21^−/−^Mid1*^−/y^ mice by tail vein injection. Mice were injected with adenovirus on day 0 and sacrificed on day 7. NFAT-Luc (1665) was purchased from Vector Biolabs. *Mid1*, *Dream* and *Ppp2ca* RNAi adenoviruses were constructed using the sequence 5′-GGCGGACAGCTGGATGATCGTG-3′, 5′-GGCCATCCACTTTGAGGACTT-3′ and 5′-GGTCCAATGTGTGACTTGCTG-3′, respectively. All expressed constructs used in this study were confirmed by sequencing.

### Cell culture and transfection

HEK293T and HepG2 (ATCC) cells were respectively cultured in DMEM and MEM containing 10% FBS (HyClone) and 100 mg/ml penicillin-streptomycin. All cell lines were routinely tested for mycoplasma using a PCR detection kit (Sigma, MP0035). Mouse primary hepatocytes were isolated as previously described^62^ and cultured in M199 medium containing 2% FBS, 0.2% BSA until attached, then continuously cultured in M199 medium without FBS. Cells were transfected with Lipofectamine 2000 (Thermo Fisher Scientific) following the manufacturer's protocol.

### Luciferase assay

For siRNA library screening of E3 ligases, HepG2 cells were transfected with 4 × CLEAR-Luc, RSV-Luc and siRNAs from human ON-TARGET plus siRNA libraries (G-105615, G-105625 and G-105635, Dharmacon). Luciferase assays were performed after 48-h transfection using the Dual Luciferase Reporter Assay System (Promega) and normalized to co-transfected RSV-Luc activity. For reporter studies, mouse primary hepatocytes were infected by WT or mutated *Mid1*-Luc, *PDYN*-Luc or NFAT-Luc (2 pfu per cell), measured after 48 h and normalized to co-infected β-galactosidase activity.

### Calcium imaging

Mouse primary hepatocytes were plated on glass coverslips and loaded with 5 μM Fura-2 acetoxymethyl ester (Molecular Probes) in the presence of 0.025% (w/v) pluronic F127 (Sigma-Aldrich) in Media 199 (Corning) for 30 min. Coverslips were mounted on a laminar flow perfusion chamber (Warner Instruments) and perfused with Media 199 or a solution of chemicals in Media 199. Images of Fura-2-loaded cells were collected with a cooled CCD camera, whereas the excitation wavelength was alternated between 340 and 380 nm. The ratio of fluorescence intensity at the two excitation wavelengths was calculated after subtracting the background fluorescence. Images were collected and analyzed using the MetaFluor software package. Graphs represent average responses from groups of 30-40 individual cells from representative single experiments. All experiments were repeated at least three times with similar results.

### *In vitro* dephosphorylation assay

The assay was performed as previously reported^54^ with modification. HA-tagged TFEB and FLAG-tagged PPP2CA were purified from mouse primary hepatocytes. TFEB-HA was incubated with PPP2CA-FLAG plus phosphatase-depleted protective lysate at 30 °C for 30 min.

### Animals

The *Fgf21*^−/−^ mice^49^ were kindly provided by Dr Nobuyuki Itoh (Kyoto University Graduate School of Pharmaceutical Sciences, Japan) and Morichika Konishi (Kobe Pharmaceutical University, Japan). The *Mid1*^−/−^ mice^63,64^ were from Dr Zhiqi Xiong (Institute of Neuroscience, Chinese Academy of Sciences, China) and originally from Dr Alan Ashworth (Institute of Cancer Research, UK). Double-knockout male mice (*Fgf21^−/−^Mid1*^−/y^) were generated by mating female *Fgf21*^+/−^*Mid1*^+/−^ mice with male *Fgf21*^+/−^*Mid1*^+/y^mice. All mice used were maintained in a C57BL/6 strain background. Genotyping was performed using PCR methods as previously described^49,63,64^ and further confirmed by qPCR and immunoblots. Mice were housed in colony cages with a 12-h light/dark cycle in a temperature-controlled environment. All animal experiments were approved by the Animal Care and Use Committee at Tsinghua University.

### *In vivo* analysis, tissue histology and immunostaining

TG levels (TR0100, Sigma) in liver tissue and plasma FGF21 (MF2100, R&D) were measured according to the manufacturer's instructions. For tissue histology, mouse tissues were fixed in 4% paraformaldehyde (PFA) and paraffin embedded. Sections (5 μm) were used for hematoxylin and eosin staining. For liver tissue immunostaining, mouse livers were dissected, fixed with buffered 4% PFA overnight at 4 °C, cryoprotected in 30% sucrose solution overnight, and finally embedded in OCT (Sakura). For immunostaining of mouse primary hepatocytes, cells were fixed in 4% PFA for 10 min. Fixed cells or cryostat sections (8 μm) were permeabilized in 0.2% Triton X-100 or 40 μM Digitonin for 5 min and blocked in 5% BSA for 1 h, and then incubated with BODIPY, or the appropriate primary and secondary antibodies.

### Subcellular fractionation of liver tissues

Subcellular fractionation was performed as described previously with modification^65^. In brief, fresh mouse liver tissues were homogenized in lysis buffer (10 mM Tris-HCl, pH 7.4; 10 mM NaCl; 0.2% NP-40) with protease inhibitor cocktail. The supernatant after centrifugation at 15 000 rpm for 10 min at 4 °C was the cytosolic fraction, whereas the pellet (the nuclear fraction) was resuspended in nuclear lysis buffer (50 mM HEPES, pH 7.4; 150 mM NaCl; 1% SDS) with protease inhibitor cocktail.

### Quantitative PCR

Total RNA from mouse liver was extracted using a Total RNA Purification kit (TR01-150, GeneMark). cDNA was obtained with a RevertAid First Strand cDNA Synthesis kit (K1622, Thermo Scientific). RNA levels were measured with a LightCycler 480 II (Roche) as previously described^62^. The following primers were used for qPCR:

*Acox1*-foward: 5′-CTGCCAAGGGACTCCAGAGCAGCT-3′, *Acox1*-reverse: 5′-GACATGGACACATCCACCATGCAG-3′

*Actin*-forward: 5′-GTCCACCCCGGGGAAGGTGA-3′, *Actin*-reverse: 5′-AGGCCTCAGACCTGGGCCATT-3′

*Atp6v0e1*-froward: 5′-GCATACCACGGCCTTACTGT-3′, *Atp6v0e1*-reverse: 5′-GAGGATTGAGCTGTGCCAGA-3′

*Atp6v1a*-forward: 5′-GAGCCCGGGCAGGTAAAT-3′, *Atp6v1a*-reverse: 5′-AATTTCTCCAACCAGCTCGC-3′

*Atp6v1h*-forward: 5′-ACTCCCCGAGGCTATCCAG-3′, *Atp6v1h*-reverse: 5′-CACGAACTTCAGCAGCCTTG-3′

*Clcn7*-forwad: 5′-CACGGCCAGGGAAGTAATGAG-3′, *Clcn7*-reverse: 5′-CGCAGGATCAAGCCTTGGAG-3′

*Cpt1α*-forward: 5′-CTGAGCCATGAAGCCCTCAA-3′, *Cpt1*-reverse: 5′-CACACCCACCACCACGATAA-3′

*Ctsa*-forward: 5′-CAGCCCCTTCCAACTACCTC-3′, *Ctsa*-reverse: 5′-CCGTTGTAGAGCAGGATCTGG-3′

*Ctsb*-forward: 5′-CTTAGGAGTGCACGGGAGAG-3′, *Ctsb*-reverse: 5′-CTTGTCATGGGCACTGGTCA-3′

*Ctsd*-forward: 5′-TACTCCATGCAGTCATCGCC-3′, *Ctsd*-reverse: 5′-GACGACTGTGAAACACTGCG-3′

*Ctsf*-forward: 5′-TGGCTCCACTCTTCAAGGAC-3′, *Ctsf*-reverse: 5′-ATCCCATACTGAGCTGTGCC-3′

*Lamp1*-forward: 5′-GCCCACAAACCCCACTGTAT-3′, *Lamp1*-reverse: 5′-TTTGGGCTGATGTTGAACGC-3′

*Mcoln1*-forward: 5′-TCATTGCACTCATCACCGGC-3′, *Mcoln1*-reverse: 5′-CCAGATGTGGGGCTATCCTG-3′

*Mid1* isoform 1-forward: 5′-TCCTTGGAGGACCCACAGGTTT-3′, *Mid1* isoform 2-forward: 5′-CAACATTGGAGACACTGATAGCTG-3′, *Mid1*-reverse: 5′-CAGACAAATAGGACAGGTCAGCT-3′

*Pgc1α*-forward: 5′-GACTTGGATACAGACAGCTTTCTGG-3′, *Pgc1α*-reverse: 5′-GTTTGCCTCATTCTCTTCATCTATC-3′

*Pparα*-forward: 5′-TCTCCACGTTCCAGCCCTTCCTCA-3′, *Pparα*-reverse: 5′-TTCACATGCGTGAACTCCGTAGTG-3′

*Sqstm1*-forward: 5′-AAGTCAGCAAACCTGACGGG-3′, *Sqstm1*-reverse: 5′-AATCAGCCGGGGATCAGC-3′

*Vps11*-forward: 5′-AAGGAGCCGCTGGGTAATGAT-3′, *Vps11*-reverse: 5′-TTGTAGGCCTGGAACCCTGTA-3′

*Vps18*-forward: 5′-GCCCGCACCGTGTACATTAT-3′, *Vps18*-reverse: 5′-TGCGAAGTTCCTCATCCTCC-3′

*Wipi1*-forward: 5′-AGGCCGGTTACAAGCTGTTT-3′, *Wipi1*-reverse: 5′-ACGTTCATCTGCCGAGGTTT-3′.

### Immunoblotting and immunoprecipitation

Assays were performed as described previously^62^. S6K, pS6K, CREB, tubulin, HA and Flag antibodies were as previously described^62^. Other antibodies were purchased as follows: anti-LC3 (PM036), MBL International; anti-P62 (610833), BD Biosciences; anti-AMPKα (2532S), anti-pAMPKα (2535S), anti-ULK1 (8054S), anti-pULK1 (Ser555) (5869S), anti-ERK (4695S), anti-pERK (4370S), anti-PLCγ1 (5690P), anti-pPLCγ1 (Tyr783) (14008S), Cell Signaling Technology; anti-TFEB (A303-673A), anti-InsP3R1 (A302-158A), Bethyl Laboratories; anti-PPP1CA (AYA361), anti-PPP1CB (AYA1025), anti-PPP1CC (AYA1169), ayaBIO; anti-calcineurin (A1063), ABclonal Technology; anti-FGFR1 (GTX107393), anti-β-Klotho (GTX45558), anti-InsP3R2 (GTX54772), GeneTex; anti-ASB3 (AP16752A), Abgent; anti-LAMP1 (L1418), anti-MID1 (M2198), Sigma-Aldrich; anti-UBR5 (ab134089), Abcam; anti-GFP (MMS-118P), Covance; anti-Ub (SC-8017), anti-PPP2CA (SC-80665), anti-DREAM (SC-9142), Santa Cruz.

### Chromatin immunoprecipitation

ChIP was performed as described previously^66^. In brief, mouse primary hepatocytes or liver samples were treated with 1% formaldehyde to cross-link protein-DNA complexes. Immunoprecipitates of cross-linked complexes were prepared with rabbit IgG or DREAM antibody, further treated with NaCl, then incubated at 65 °C to release cross-links. DNA was purified with a PCR purification kit (AP-PCR-250G, AXYGEN) and then analyzed by qPCR to amplify *Mid1* promoter sequences. The primers for ChIP were designed as follows: 5′-GTGCAGCTTCTTCTTGTGAGAG-3′ and 5′-CTAGTTGCTGGAGGCCAAGC-3′. All signals were normalized to the input chromatin signals.

### Mass spectrometry

To identify the regulatory phosphorylation sites of TFEB by PP2A, mouse primary hepatocytes were infected with FLAG-TFEB, pre-treated with or without OA (100 nM) for 1 h and then co-incubated with or without FGF21 for another 4 h. Immunoprecipitates of FLAG-TFEB were analyzed by electrospray ionization tandem mass spectrometry on a Thermo LTQ Orbitrap instrument as previously described^62^.

### Statistical analyses

Age- and weight-matched male mice were randomly assigned to different experimental groups. The number of animals used in each experiment is stated in the corresponding figure legend. No animals were excluded from statistical analyses, and the outcome was not blinded before scoring. All studies were performed on at least three independent occasions. Results are reported as mean ± SEM. Comparison of different groups was carried out using two-tailed unpaired Student's t-test. Differences were considered statistically significant at P < 0.05.

## Author Contributions

LC, KW, JH and YW designed the study. LC, KW, AL, LJ, YZ, HD, YL and YW performed the experiments. LC, KW, LJ, JH and YW analyzed the data. LC, KW, JH and YW wrote and edited the manuscript.

## Competing Financial Interests

The authors declare no competing financial interests.

## Supplementary Information

Supplementary information, Figure S1 Correlation of FGF21 level, autophagy and lipid metabolism.

Supplementary information, Figure S2 Comparison of FGF21 signaling in young and aged mice.

Supplementary information, Figure S3 Effect of *Klb* knockdown on lysosomal function.

Supplementary information, Figure S4 Characterization of the nuclearlocalized TFEB mutant.

Supplementary information, Figure S5 Effect of PP2A on TFEB phosphorylation.

Supplementary information, Figure S6 MID1 mediates TFEB activity.

Supplementary information, Figure S7 Evaluation of cellular localization of DREAM and its mutant.

Supplementary information, Figure S8 Evaluation of PLCγ activation.
