# Supplementary material for: Fasting-induced hormonal regulation of lysosomal function
Source: Cell Res. 2017 Apr 4;27(6):748–63. doi: 10.1038/cr.2017.45 (PMC5518872; doi:10.1038/cr.2017.45)
Supplement: Supplementary information, Figure S1 — Correlation of FGF21 level, autophagy and lipid metabolism. [file cr201745x1.pdf]

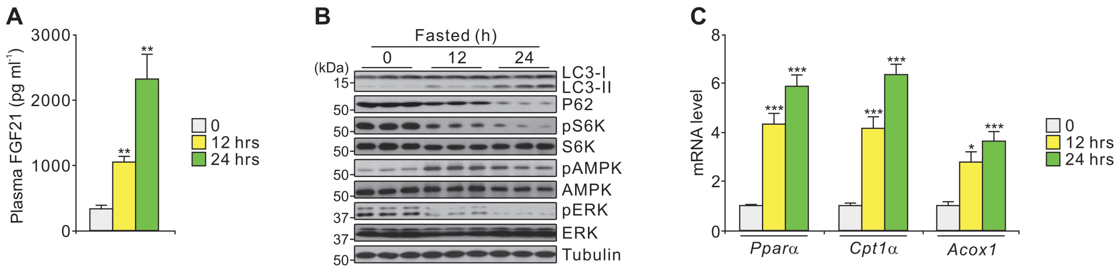

Supplementary information, Figure S1. Correlation of FGF21 level, autophagy and lipid metabolism. Plasma FGF21 levels (**A**), immunoblots (**B**) and qPCR results (**C**) showing expression levels of genes (*Pparα*, *Cpt1* and *Acox1*) involved in fatty acid β-oxidation of liver extracts from mice fed a regular diet or fasted for 12 h or 24 h. Data are shown as mean ± s.e.m. $*P < 0.05$, $**P < 0.01$, $***P < 0.001$, n = 6 mice per group.
