# Supplementary material for: Fasting-induced hormonal regulation of lysosomal function
Source: Cell Res. 2017 Apr 4;27(6):748–63. doi: 10.1038/cr.2017.45 (PMC5518872; doi:10.1038/cr.2017.45)
Supplement: Supplementary information, Figure S2 — Comparison of FGF21 signaling in young and aged mice. [file cr201745x2.pdf]

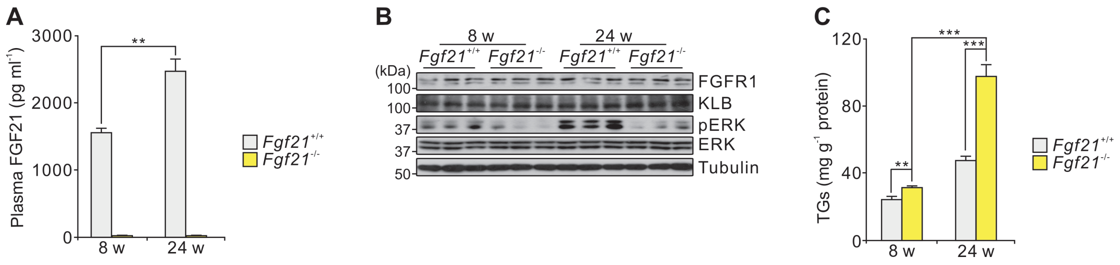

Supplementary information, Figure S2. Comparison of FGF21 signaling in young and aged mice. Plasma FGF21 levels (**A**), immunoblots (**B**) and hepatic triglyceride levels (**C**) in 8-week- or 24-week-old mice fasted for 24 h. Data are shown as mean ± s.e.m. $^{**}P < 0.01$, $^{***}P < 0.001$, n = 6 mice per group.
