# Supplementary material for: Fasting-induced hormonal regulation of lysosomal function
Source: Cell Res. 2017 Apr 4;27(6):748–63. doi: 10.1038/cr.2017.45 (PMC5518872; doi:10.1038/cr.2017.45)
Supplement: Supplementary information, Figure S3 — Effect of Klb knockdown on lysosomal function. [file cr201745x3.pdf]

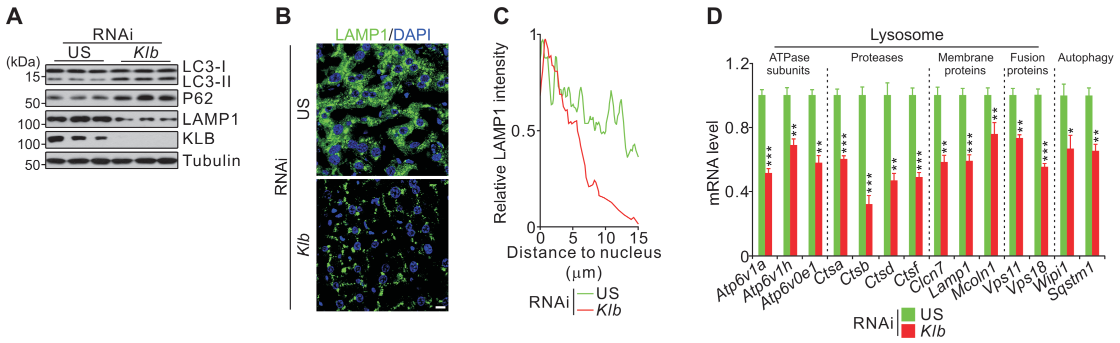

Supplementary information, Figure S3. Effect of *Klb* knockdown on lysosomal function. Immunoblots (**A**), LAMP1 staining (**B**) and quantification (**C**), and qPCR results (**D**) showing the effect of *Klb* knockdown in mice fasted for 24 h. Scale bar, 10 μm. Data are shown as mean ± s.e.m. **P* < 0.05, ***P* < 0.01, ****P* < 0.001, n = 8 mice per group.
