# Supplementary material for: Fasting-induced hormonal regulation of lysosomal function
Source: Cell Res. 2017 Apr 4;27(6):748–63. doi: 10.1038/cr.2017.45 (PMC5518872; doi:10.1038/cr.2017.45)
Supplement: Supplementary information, Figure S4 — Characterization of the nuclearlocalized TFEB mutant. [file cr201745x4.pdf]

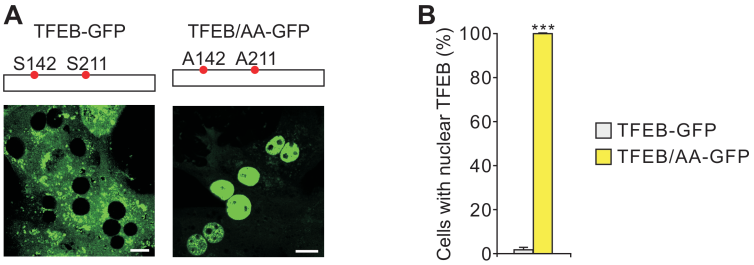

Supplementary information, Figure S4. Characterization of the nuclear-localized TFEB mutant. (**A**) Schematic (top panel) and images (bottom panel) showing the phosphorylation sites and cellular localization of wildtype and mutated TFEB in mouse primary hepatocytes. (**B**) Quantification of nuclear translocation of wildtype and mutated TFEB in mouse primary hepatocytes. Scale bars, 10 μm.
