# Supplementary material for: Fasting-induced hormonal regulation of lysosomal function
Source: Cell Res. 2017 Apr 4;27(6):748–63. doi: 10.1038/cr.2017.45 (PMC5518872; doi:10.1038/cr.2017.45)
Supplement: Supplementary information, Figure S5 — Effect of PP2A on TFEB phosphorylation. [file cr201745x5.pdf]

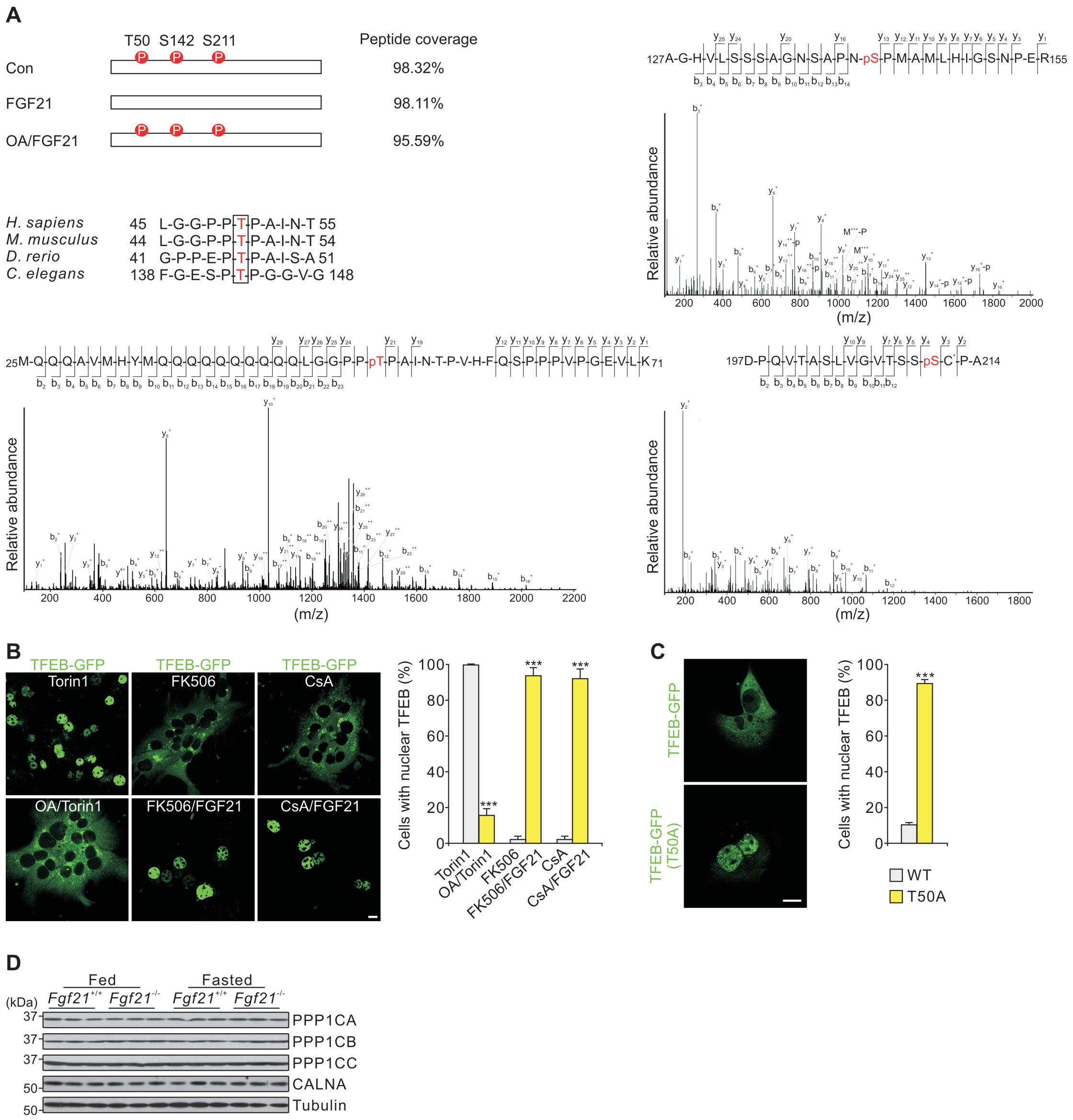

Supplementary information, Figure S5. Effect of PP2A on TFEB phosphorylation. (**A**) Schematic showing phosphorylation sites of TFEB identified by mass spectrometry and peptide coverage (top left panel), amino acid sequence alignment of TFEB orthologs with the threonine phosphorylation site boxed (middle left panel), and MS/MS spectra of the phosphorylated peptides. The labeled peaks show the masses of *y* or *b* ions of the phosphorylated peptide. C* indicates carbamidomethyl cysteine. To

identify the regulatory dephosphorylation sites of TFEB by PP2A, mouse primary hepatocytes were infected with FLAG-TFEB, pre-treated with or without okadaic acid (100 nM) for 1 h and then co-incubated with or without FGF21 for another 4 h. The total cell lysate was immunoprecipitated by anti-FLAG M2 affinity gel and further analyzed by electrospray ionization tandem MS. (**B**) Representative images and quantification showing the effect of different chemical treatments on cellular localization of TFEB in mouse primary hepatocytes. Mouse primary hepatocytes were incubated with okadaic acid (OA, 100 nM), Torin1 (250 nM), FK506 (5 μM) or Cyclosporin A (CsA, 10 μM) for 1 h before 4 h FGF21 (50 ng ml^{-1}) or 1 h Torin1 treatment. (**C**) Cellular localization and quantification of wildtype and mutated TFEB (alanine mutation at threonine 50) in HepG2 cells. (**D**) Immunoblots showing the levels of protein phosphatases in liver extracts from $Fgf21^{+/+}$ and $Fgf21^{-/-}$ mice fed or fasted for 24 h. Scale bars, 10 μm.
