# Supplementary material for: Fasting-induced hormonal regulation of lysosomal function
Source: Cell Res. 2017 Apr 4;27(6):748–63. doi: 10.1038/cr.2017.45 (PMC5518872; doi:10.1038/cr.2017.45)
Supplement: Supplementary information, Figure S6 — MID1 mediates TFEB activity. [file cr201745x6.pdf]

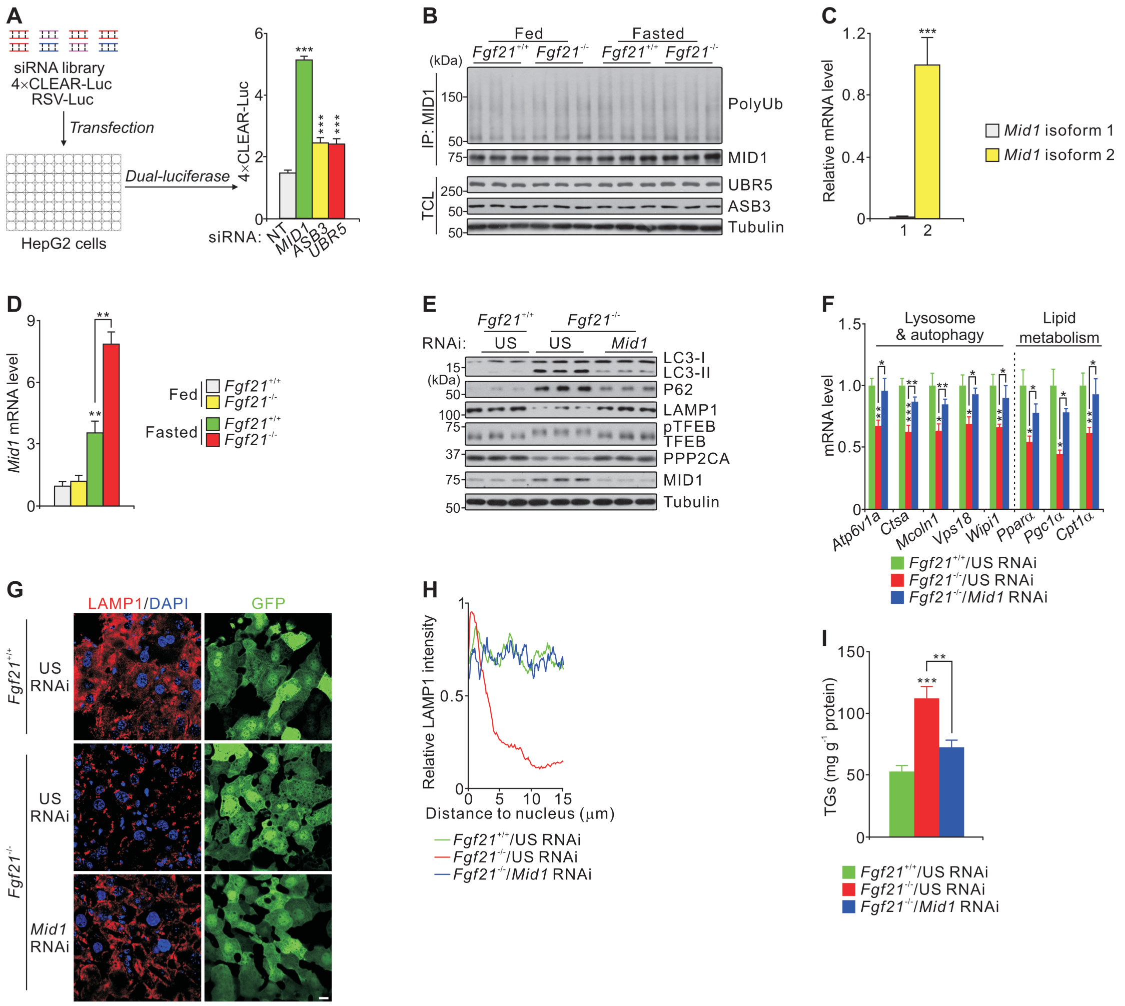

Supplementary information, Figure S6. MID1 mediates TFEB activity. (**A**) siRNA library screening for E3 ligases that modulate the activity of TFEB (measured by 4×CLEAR-Luc activity) in HepG2 cells. (**B**) Immunoblots showing the levels of UBR5, ASB3 and ubiquitinated MID1 in liver extracts from *Fgf21*$^{+/+}$ and *Fgf21*$^{-/-}$ mice fed or fasted for 24 h. (**C**) qPCR results showing relative hepatic levels of *Mid1* isoform 1 and isoform 2 in wildtype mice fed with a regular diet. (**D**) qPCR results in liver extracts from *Fgf21*$^{+/+}$ and *Fgf21*$^{-/-}$ mice fed or fasted for 24 h. (**E-I**), Immunoblots (**E**), qPCR results (**F**), LAMP1 staining (**G**) and quantification (**H**), and hepatic triglyceride content (**I**) showing the effect of *Mid1* knockdown in *Fgf21*$^{-/-}$ mice fasted for 24

h. Scale bar, 10 μm. Data are shown as mean ± s.e.m. *P < 0.05, **P < 0.01, ***P < 0.001, n = 8 mice or 5 replicates for HepG2 cells per group.
