# Supplementary material for: Fasting-induced hormonal regulation of lysosomal function
Source: Cell Res. 2017 Apr 4;27(6):748–63. doi: 10.1038/cr.2017.45 (PMC5518872; doi:10.1038/cr.2017.45)
Supplement: Supplementary information, Figure S7 — Evaluation of cellular localization of DREAM and its mutant. [file cr201745x7.pdf]

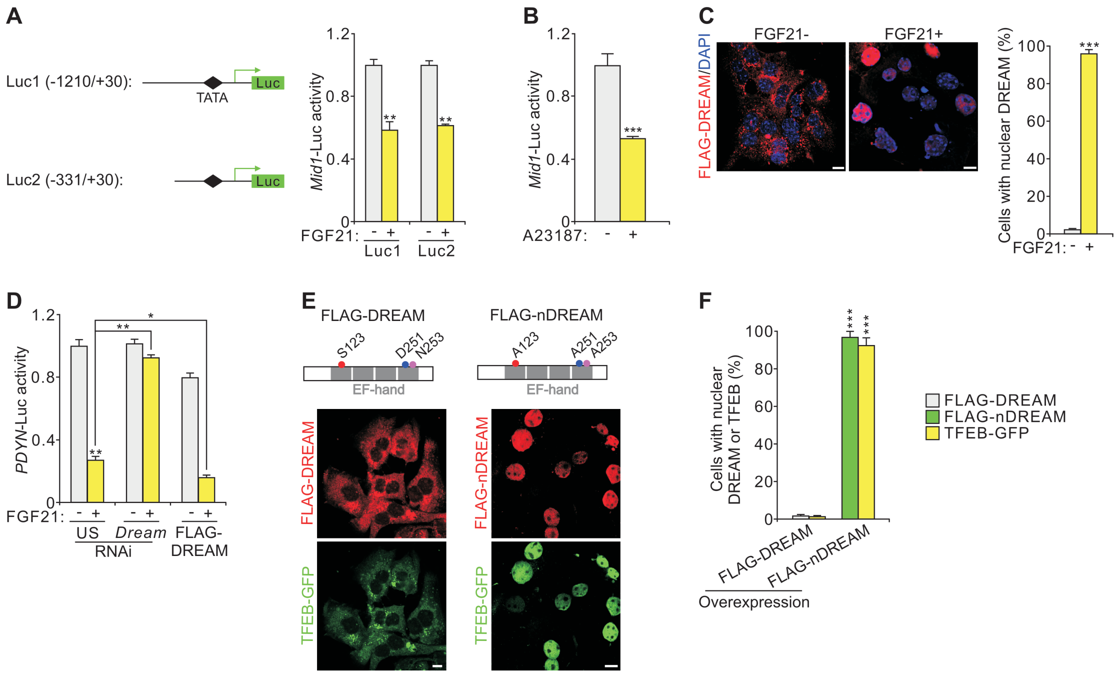

Supplementary information, Figure S7. Evaluation of cellular localization of DREAM and its mutant. (**A**) Schematic showing different truncations of *Mid1*-Luc, location of the TATA box of the mouse *Mid1* gene and the transcriptional start site (green arrow) (left panel), and reporter activity of *Mid1*-Luc in presence or absence of FGF21 (right panel). (**B**) Effect of the calcium ionophore A23187 (10 μM) on *Mid1*-Luc (-331/+30) activity. (**C**) Nuclear shuttling of FLAG-DREAM after FGF21 (50 ng ml^{-1}) incubation for 1 h in mouse primary hepatocytes. (**D**) Effect of FLAG-DREAM overexpression or *Dream* knockdown on *PDYN*-Luc activity in presence or absence of FGF21. (**E**) Schematic (top panel) and images (bottom panel) showing the critical sites and cellular localization of wildtype and mutated DREAM (nDREAM, triple alanine mutations at serine 123, aspartic acid 251 and asparagine 253) in mouse primary hepatocytes. Effect of wildtype and nDREAM on TFEB-GFP localization is also shown. (**F**) Quantification of nuclear translocation of

DREAM, nDREAM or TFEB. Scale bars, 10 μm. Data are shown as mean ± s.e.m. *P < 0.05, **P < 0.01, ***P < 0.001, n = 5 replicates per group.
