# Supplementary material for: Fasting-induced hormonal regulation of lysosomal function
Source: Cell Res. 2017 Apr 4;27(6):748–63. doi: 10.1038/cr.2017.45 (PMC5518872; doi:10.1038/cr.2017.45)
Supplement: Supplementary information, Figure S8 — Evaluation of PLCγ activation. [file cr201745x8.pdf]

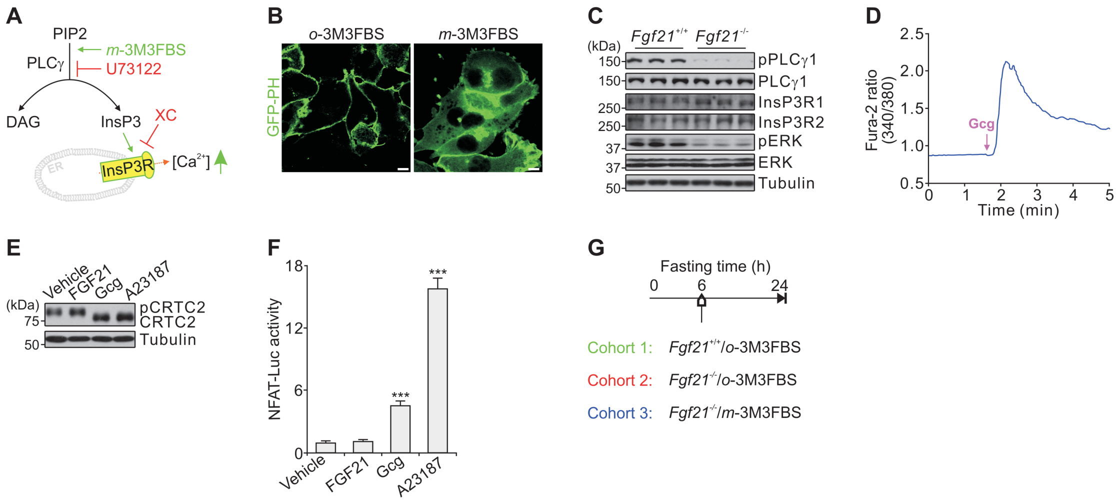

Supplementary information, Figure S8. Evaluation of PLCγ activation. (**A**) Diagram showing inhibitor or activator targets. (**B**) Evaluation of PLC activation, judged by localization of GFP-PH, in mouse primary hepatocytes treated with the PLC activator *m*-3M3FBS. Hepatocytes were incubated with *m*-3M3FBS (10 μM) or its inactive analog *o*-3M3FBS (10 μM) for 30 min. (**C**) Immunoblots of liver extracts from *Fgf21*$^{+/+}$ and *Fgf21*$^{-/-}$ mice fasted for 24 h. (**D**) Effect of Glucagon (Gcg, 100 nM) on calcium mobilization in mouse primary hepatocytes. (**E-F**) Effect of FGF21, Gcg and A23187 on CRTC2 dephosphorylation (**E**) and NFAT-Luc activity (**F**) in mouse primary hepatocytes. Hepatocytes were incubated with FGF21 (50 ng ml^{-1}), Gcg (100 nM) or A23187 (10 μM) for 4 h. (**G**) Schematic showing the protocol for 3M3FBS treatment. After 6 h fasting, mice were given an intraperitoneal injection of 6 mg kg^{-1} *o*-3M3FBS or *m*-3M3FBS. Analyses were performed after a further 18 h. Scale bars, 10 μm.
